# Methyl *N*-(2,3-dichloro­phen­yl)succinamate

**DOI:** 10.1107/S1600536810014844

**Published:** 2010-04-28

**Authors:** B. Thimme Gowda, Sabine Foro, B. S. Saraswathi, Hartmut Fuess

**Affiliations:** a Department of Chemistry, Mangalore University, Mangalagangotri 574 199, Mangalore, India; b Institute of Materials Science, Darmstadt University of Technology, Petersenstrasse 23, D-64287 Darmstadt, Germany

## Abstract

The asymmetric unit of the title compound, C_11_H_11_Cl_2_NO_3_, contains two independent mol­ecules. In both the molecules, the H atoms of the adjacent  –CH_2_ groups of the acid segments orient themselves away from the amide O and the carbonyl O atoms. The C=O and O—CH_3_ bonds of the ester group are in *syn* positions with respect to each other. In the crystal, the mol­ecules are linked into infinite chains through inter­molecular N—H⋯O hydrogen bonds.

## Related literature

For related structures, see: Gowda *et al.* (2009**a* ▶,b* ▶); Saraswathi *et al.* (2010 ▶).
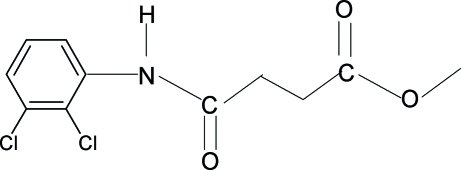


## Experimental

### 

#### Crystal data

C_11_H_11_Cl_2_NO_3_
*M* *_r_* = 276.11
Triclinic, 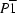

*a* = 4.7356 (5) Å
*b* = 15.868 (1) Å
*c* = 17.158 (2) Å
α = 80.748 (8)°
β = 88.869 (8)°
γ = 82.350 (8)°
*V* = 1261.2 (2) Å^3^
*Z* = 4
Mo *K*α radiation
μ = 0.51 mm^−1^
*T* = 299 K
0.30 × 0.12 × 0.06 mm

#### Data collection

Oxford Diffraction Xcalibur diffractometer with a Sapphire CCD detector
Absorption correction: multi-scan (*CrysAlis RED*; Oxford Diffraction, 2009 ▶) *T*_min_ = 0.862, *T*_max_ = 0.970
8311 measured reflections
4564 independent reflections
3398 reflections with *I* > 2σ(*I*)
*R*_int_ = 0.019

#### Refinement

*R*[*F* ^2^ > 2σ(*F* ^2^)] = 0.054
*wR*(*F* ^2^) = 0.101
*S* = 1.16
4564 reflections
313 parameters
14 restraints
H atoms treated by a mixture of independent and constrained refinement
Δρ_max_ = 0.26 e Å^−3^
Δρ_min_ = −0.25 e Å^−3^

### 

Data collection: *CrysAlis CCD* (Oxford Diffraction, 2009 ▶); cell refinement: *CrysAlis RED* (Oxford Diffraction, 2009 ▶); data reduction: *CrysAlis RED*; program(s) used to solve structure: *SHELXS97* (Sheldrick, 2008 ▶); program(s) used to refine structure: *SHELXL97* (Sheldrick, 2008 ▶); molecular graphics: *PLATON* (Spek, 2009 ▶); software used to prepare material for publication: *SHELXL97*.

## Supplementary Material

Crystal structure: contains datablocks I, global. DOI: 10.1107/S1600536810014844/ng2762sup1.cif

Structure factors: contains datablocks I. DOI: 10.1107/S1600536810014844/ng2762Isup2.hkl

Additional supplementary materials:  crystallographic information; 3D view; checkCIF report

## Figures and Tables

**Table 1:** Hydrogen-bond geometry (Å, °)

*D*—H⋯*A*	*D*—H	H⋯*A*	*D*⋯*A*	*D*—H⋯*A*
N1—H1*N*⋯O1^i^	0.85 (1)	2.08 (1)	2.899 (3)	162 (3)
N2—H2*N*⋯O4^ii^	0.86 (1)	2.06 (2)	2.880 (3)	159 (3)

Symmetry codes: (i) 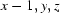; (ii) 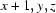.

**Fig. 1.**
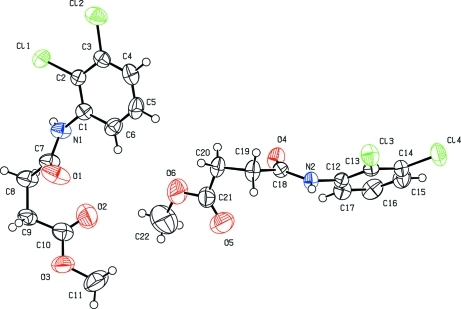
Molecular structure of the title compound, showing the atom labelling scheme. The displacement ellipsoids are drawn at the 50% probability level. The H atoms are represented as small spheres of arbitrary radii.

**Fig. 2.**
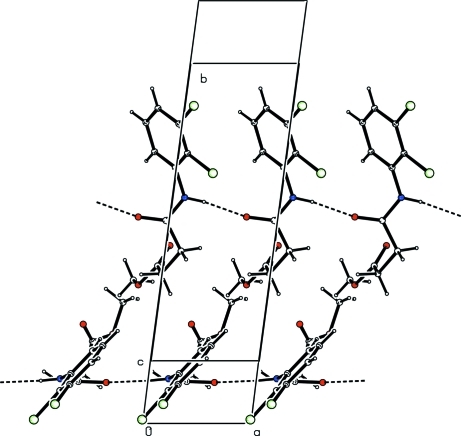
Molecular packing of the title compound with hydrogen bonding shown as dashed lines.

## supplementary crystallographic information

### Comment

As a part of studying the effect of ring and side chain substitutions on the structures of biologically significant compounds (Gowda *et al.*, 2009*a,b*; Saraswathi *et al.*, 2010). the crystal structure of *N*-(3,4-dichlorophenyl)methylsuccinamate (I), systematic name: 3-[(3,4-dichloro)-aminocarbonyl]propionate has been determined. The asymmetric unit of the structure contains 2 independent molecules. The conformations of N—H and C=O bonds in the amide segments of the structure are *anti* to each other. Further, the conformation of the amide O atom and the carbonyl O atom of the ester segment are *anti* to the H atoms attached to the adjacent C atoms (Fig.1), similar to that observed in *N*-(3,5-dichlorophenyl)-methylsuccinamate (Saraswathi *et al.*, 2010) and *N*-(4-chlorophenyl)-methylsuccinamate (Gowda *et al.*, 2009*b*). The C=O and O–CH_2_ bonds of the ester group are in *syn* position to each other. The N—H···O intermolecular hydrogen bonds pack the mpolecules into infinite chains in the structure (Table 1, Fig.2).

### Experimental

The solution of succinic anhydride (0.02 mol) in toluene (25 ml) was treated dropwise with the solution of 3,4-dichloroaniline (0.02 mol) also in toluene (20 ml) with constant stirring. The resulting mixture was stirred for about one hour and set aside for an additional hour at room temperature for the completion of reaction. The mixture was then treated with dilute hydrochloric acid to remove the unreacted 3,4-dichloroaniline. The resultant solid *N*-(3,4-dichlorophenyl)succinamic acid was filtered under suction and washed thoroughly with water to remove the unreacted succinic anhydride and succinic acid. It was recrystallized to constant melting point from methanol. Pure *N*-(3,4-dichlorophenyl)succinamic acid in methanol was refluxed with 2 ml of conc. sulfuric acid for two hours and was subjected to slow evaporation. The resulting *N*-(3,4-dichlorophenyl)methylsuccinamate was recrystallised from methanol. The purity of the compound was checked and characterized by its infrared and NMR spectra.

Needle like colourless single crystals used in X-ray diffraction studies were grown in methanol solution by slow evaporation at room temperature.

### Refinement

The H atoms of the NH groups were located in a difference map and their position refined with N—H = 0.86 (1) %A. The other H atoms were positioned with idealized geometry using a riding model with C—H = 0.93–0.97 Å. All H atoms were refined with isotropic displacement parameters (set to 1.2 times of the U_eq_ of the parent atom). The U^ij^ components of C11 and C22 were restrained to approximate isotropic behavoir.

### Crystal data

**Table:**

C_11_H_11_Cl_2_NO_3_	*Z* = 4
*M**_r_* = 276.11	*F*(000) = 568
Triclinic, *P*1	*D*_x_ = 1.454 Mg m^−^^3^
Hall symbol: -P 1	Mo *K*α radiation, λ = 0.71073 Å
*a* = 4.7356 (5) Å	Cell parameters from 3119 reflections
*b* = 15.868 (1) Å	θ = 2.5–27.9°
*c* = 17.158 (2) Å	µ = 0.51 mm^−^^1^
α = 80.748 (8)°	*T* = 299 K
β = 88.869 (8)°	Needle, colourless
γ = 82.350 (8)°	0.30 × 0.12 × 0.06 mm
*V* = 1261.2 (2) Å^3^	

### Data collection

**Table:**

Oxford Diffraction Xcalibur diffractometer with a Sapphire CCD detector	4564 independent reflections
Radiation source: fine-focus sealed tube	3398 reflections with *I* > 2σ(*I*)
graphite	*R*_int_ = 0.019
Rotation method data acquisition using ω and φ scans	θ_max_ = 25.3°, θ_min_ = 2.6°
Absorption correction: multi-scan (*CrysAlis RED*; Oxford Diffraction, 2009)	*h* = −5→4
*T*_min_ = 0.862, *T*_max_ = 0.970	*k* = −18→19
8311 measured reflections	*l* = −20→20

### Refinement

**Table:**

Refinement on *F*^2^	Primary atom site location: structure-invariant direct methods
Least-squares matrix: full	Secondary atom site location: difference Fourier map
*R*[*F*^2^ > 2σ(*F*^2^)] = 0.054	Hydrogen site location: inferred from neighbouring sites
*wR*(*F*^2^) = 0.101	H atoms treated by a mixture of independent and constrained refinement
*S* = 1.16	*w* = 1/[σ^2^(*F*_o_^2^) + (0.0142*P*)^2^ + 1.3931*P*] where *P* = (*F*_o_^2^ + 2*F*_c_^2^)/3
4564 reflections	(Δ/σ)_max_ < 0.001
313 parameters	Δρ_max_ = 0.26 e Å^−^^3^
14 restraints	Δρ_min_ = −0.25 e Å^−^^3^

### Special details

**Table:**

Geometry. All esds (except the esd in the dihedral angle between two l.s. planes) are estimated using the full covariance matrix. The cell esds are taken into account individually in the estimation of esds in distances, angles and torsion angles; correlations between esds in cell parameters are only used when they are defined by crystal symmetry. An approximate (isotropic) treatment of cell esds is used for estimating esds involving l.s. planes.
Refinement. Refinement of *F*^2^ against ALL reflections. The weighted *R*-factor wR and goodness of fit *S* are based on *F*^2^, conventional *R*-factors *R* are based on *F*, with *F* set to zero for negative *F*^2^. The threshold expression of *F*^2^ > σ(*F*^2^) is used only for calculating *R*-factors(gt) etc. and is not relevant to the choice of reflections for refinement. *R*-factors based on *F*^2^ are statistically about twice as large as those based on *F*, and *R*- factors based on ALL data will be even larger.

### Fractional atomic coordinates and isotropic or equivalent isotropic displacement parameters (Å^2^)

**Table:**

	*x*	*y*	*z*	*U*_iso_*/*U*_eq_	
Cl1	−0.00812 (19)	−0.02952 (6)	0.23112 (5)	0.0573 (3)	
Cl2	0.1692 (2)	−0.01708 (6)	0.40431 (5)	0.0666 (3)	
O1	0.6129 (5)	0.1051 (2)	0.04979 (15)	0.0817 (9)	
O2	0.3278 (7)	0.2834 (2)	−0.0451 (2)	0.0989 (11)	
O3	0.6122 (6)	0.27365 (19)	−0.14800 (15)	0.0789 (9)	
N1	0.1836 (5)	0.10541 (19)	0.10772 (16)	0.0474 (7)	
H1N	0.008 (3)	0.101 (2)	0.1013 (19)	0.057*	
C1	0.2772 (6)	0.1062 (2)	0.18535 (19)	0.0406 (7)	
C2	0.1959 (6)	0.04796 (19)	0.24829 (18)	0.0375 (7)	
C3	0.2808 (7)	0.0521 (2)	0.32437 (18)	0.0433 (8)	
C4	0.4535 (7)	0.1112 (2)	0.3378 (2)	0.0543 (9)	
H4	0.5130	0.1129	0.3888	0.065*	
C5	0.5375 (7)	0.1676 (2)	0.2755 (2)	0.0580 (10)	
H5	0.6564	0.2072	0.2845	0.070*	
C6	0.4491 (7)	0.1667 (2)	0.1997 (2)	0.0501 (9)	
H6	0.5040	0.2065	0.1582	0.060*	
C7	0.3556 (7)	0.1087 (2)	0.04437 (19)	0.0503 (9)	
C8	0.2087 (7)	0.1151 (3)	−0.03415 (19)	0.0586 (10)	
H8A	0.0246	0.1500	−0.0333	0.070*	
H8B	0.1766	0.0580	−0.0423	0.070*	
C9	0.3833 (7)	0.1545 (2)	−0.10209 (19)	0.0546 (9)	
H9A	0.5653	0.1187	−0.1038	0.065*	
H9B	0.2846	0.1559	−0.1514	0.065*	
C10	0.4338 (8)	0.2446 (3)	−0.0942 (2)	0.0581 (10)	
C11	0.6685 (11)	0.3620 (3)	−0.1463 (3)	0.0965 (16)	
H11A	0.4931	0.4004	−0.1536	0.116*	
H11B	0.7986	0.3784	−0.1879	0.116*	
H11C	0.7506	0.3651	−0.0963	0.116*	
Cl3	1.3412 (2)	0.60860 (6)	0.49659 (5)	0.0574 (3)	
Cl4	1.0827 (3)	0.78824 (7)	0.53909 (6)	0.0770 (3)	
O4	0.7056 (4)	0.51729 (14)	0.32203 (15)	0.0534 (6)	
O5	1.0323 (8)	0.4666 (2)	0.15611 (19)	0.1093 (12)	
O6	0.7887 (8)	0.3579 (2)	0.15546 (19)	0.1027 (11)	
N2	1.1105 (5)	0.56608 (16)	0.35184 (15)	0.0382 (6)	
H2N	1.293 (2)	0.5544 (19)	0.3550 (18)	0.046*	
C12	0.9873 (6)	0.64607 (18)	0.37103 (17)	0.0344 (7)	
C13	1.0824 (6)	0.67383 (19)	0.43758 (17)	0.0362 (7)	
C14	0.9662 (7)	0.7527 (2)	0.45638 (19)	0.0459 (8)	
C15	0.7541 (8)	0.8036 (2)	0.4107 (2)	0.0570 (10)	
H15	0.6754	0.8563	0.4239	0.068*	
C16	0.6598 (8)	0.7758 (2)	0.3453 (2)	0.0591 (10)	
H16	0.5158	0.8100	0.3143	0.071*	
C17	0.7756 (7)	0.6981 (2)	0.3251 (2)	0.0482 (8)	
H17	0.7111	0.6804	0.2802	0.058*	
C18	0.9643 (6)	0.5063 (2)	0.33015 (17)	0.0368 (7)	
C19	1.1431 (6)	0.4234 (2)	0.3189 (2)	0.0484 (9)	
H19A	1.2006	0.3910	0.3703	0.058*	
H19B	1.3143	0.4366	0.2902	0.058*	
C20	0.9884 (7)	0.3681 (2)	0.2746 (2)	0.0492 (9)	
H20A	1.0970	0.3112	0.2790	0.059*	
H20B	0.8045	0.3618	0.2992	0.059*	
C21	0.9442 (8)	0.4044 (3)	0.1895 (2)	0.0625 (10)	
C22	0.7374 (15)	0.3858 (4)	0.0715 (3)	0.146 (2)	
H22A	0.9162	0.3861	0.0441	0.175*	
H22B	0.6340	0.4428	0.0630	0.175*	
H22C	0.6281	0.3470	0.0517	0.175*	

### Atomic displacement parameters (Å^2^)

**Table:**

	*U*^11^	*U*^22^	*U*^33^	*U*^12^	*U*^13^	*U*^23^
Cl1	0.0583 (6)	0.0585 (6)	0.0607 (6)	−0.0252 (4)	0.0038 (4)	−0.0126 (4)
Cl2	0.0767 (7)	0.0689 (6)	0.0473 (5)	0.0011 (5)	0.0032 (5)	0.0024 (5)
O1	0.0267 (13)	0.167 (3)	0.0525 (16)	−0.0225 (16)	−0.0005 (11)	−0.0115 (17)
O2	0.101 (3)	0.107 (3)	0.103 (3)	−0.030 (2)	0.024 (2)	−0.047 (2)
O3	0.096 (2)	0.095 (2)	0.0502 (16)	−0.0474 (18)	0.0005 (15)	0.0019 (15)
N1	0.0255 (13)	0.072 (2)	0.0448 (16)	−0.0129 (14)	−0.0023 (12)	−0.0027 (14)
C1	0.0277 (16)	0.0483 (19)	0.0453 (19)	−0.0021 (14)	−0.0011 (14)	−0.0085 (15)
C2	0.0284 (16)	0.0369 (17)	0.0474 (19)	−0.0032 (13)	0.0020 (13)	−0.0086 (14)
C3	0.0401 (18)	0.0447 (19)	0.0422 (19)	0.0062 (15)	0.0024 (15)	−0.0086 (15)
C4	0.049 (2)	0.062 (2)	0.055 (2)	0.0006 (19)	−0.0076 (17)	−0.0232 (19)
C5	0.049 (2)	0.056 (2)	0.077 (3)	−0.0143 (18)	−0.0050 (19)	−0.028 (2)
C6	0.0427 (19)	0.046 (2)	0.062 (2)	−0.0095 (16)	0.0030 (17)	−0.0072 (17)
C7	0.0324 (18)	0.072 (2)	0.045 (2)	−0.0121 (17)	−0.0002 (15)	−0.0004 (17)
C8	0.0413 (19)	0.092 (3)	0.045 (2)	−0.0263 (19)	−0.0026 (16)	−0.0054 (19)
C9	0.051 (2)	0.081 (3)	0.0369 (19)	−0.0241 (19)	−0.0002 (16)	−0.0105 (18)
C10	0.048 (2)	0.086 (3)	0.041 (2)	−0.017 (2)	−0.0091 (17)	−0.006 (2)
C11	0.125 (4)	0.085 (3)	0.081 (3)	−0.052 (3)	−0.017 (3)	0.014 (3)
Cl3	0.0618 (6)	0.0576 (6)	0.0508 (5)	0.0042 (4)	−0.0204 (4)	−0.0103 (4)
Cl4	0.1133 (9)	0.0655 (6)	0.0601 (6)	−0.0138 (6)	0.0011 (6)	−0.0318 (5)
O4	0.0243 (12)	0.0540 (14)	0.0864 (18)	−0.0042 (10)	−0.0049 (11)	−0.0247 (13)
O5	0.153 (3)	0.105 (3)	0.079 (2)	−0.066 (3)	0.000 (2)	−0.002 (2)
O6	0.138 (3)	0.112 (3)	0.074 (2)	−0.059 (2)	−0.026 (2)	−0.0258 (19)
N2	0.0224 (12)	0.0433 (15)	0.0523 (16)	−0.0037 (12)	−0.0030 (12)	−0.0179 (13)
C12	0.0268 (15)	0.0348 (17)	0.0418 (17)	−0.0034 (13)	0.0033 (13)	−0.0076 (14)
C13	0.0317 (16)	0.0394 (17)	0.0375 (17)	−0.0066 (13)	0.0009 (13)	−0.0042 (14)
C14	0.055 (2)	0.0405 (19)	0.0450 (19)	−0.0094 (16)	0.0069 (16)	−0.0126 (15)
C15	0.057 (2)	0.0374 (19)	0.074 (3)	0.0035 (17)	0.010 (2)	−0.0105 (18)
C16	0.048 (2)	0.046 (2)	0.077 (3)	0.0041 (17)	−0.0098 (19)	0.0033 (19)
C17	0.0425 (19)	0.049 (2)	0.052 (2)	−0.0017 (16)	−0.0097 (16)	−0.0066 (16)
C18	0.0260 (16)	0.0455 (18)	0.0408 (17)	−0.0058 (14)	−0.0014 (13)	−0.0110 (14)
C19	0.0317 (17)	0.047 (2)	0.070 (2)	−0.0002 (15)	−0.0084 (16)	−0.0231 (17)
C20	0.0411 (19)	0.0398 (19)	0.071 (2)	−0.0047 (15)	−0.0029 (17)	−0.0211 (17)
C21	0.065 (3)	0.062 (3)	0.067 (3)	−0.015 (2)	0.001 (2)	−0.024 (2)
C22	0.186 (6)	0.181 (6)	0.086 (4)	−0.056 (5)	−0.026 (4)	−0.036 (4)

### Geometric parameters (Å, °)

**Table:**

Cl1—C2	1.725 (3)	Cl3—C13	1.724 (3)
Cl2—C3	1.734 (3)	Cl4—C14	1.734 (3)
O1—C7	1.217 (4)	O4—C18	1.222 (3)
O2—C10	1.187 (4)	O5—C21	1.181 (5)
O3—C10	1.309 (4)	O6—C21	1.315 (4)
O3—C11	1.467 (5)	O6—C22	1.454 (6)
N1—C7	1.344 (4)	N2—C18	1.348 (4)
N1—C1	1.414 (4)	N2—C12	1.411 (4)
N1—H1N	0.852 (10)	N2—H2N	0.859 (10)
C1—C2	1.387 (4)	C12—C17	1.382 (4)
C1—C6	1.393 (4)	C12—C13	1.391 (4)
C2—C3	1.388 (4)	C13—C14	1.382 (4)
C3—C4	1.372 (5)	C14—C15	1.374 (5)
C4—C5	1.368 (5)	C15—C16	1.372 (5)
C4—H4	0.9300	C15—H15	0.9300
C5—C6	1.377 (5)	C16—C17	1.376 (5)
C5—H5	0.9300	C16—H16	0.9300
C6—H6	0.9300	C17—H17	0.9300
C7—C8	1.511 (4)	C18—C19	1.504 (4)
C8—C9	1.516 (4)	C19—C20	1.514 (4)
C8—H8A	0.9700	C19—H19A	0.9700
C8—H8B	0.9700	C19—H19B	0.9700
C9—C10	1.508 (5)	C20—C21	1.490 (5)
C9—H9A	0.9700	C20—H20A	0.9700
C9—H9B	0.9700	C20—H20B	0.9700
C11—H11A	0.9600	C22—H22A	0.9600
C11—H11B	0.9600	C22—H22B	0.9600
C11—H11C	0.9600	C22—H22C	0.9600
			
C10—O3—C11	114.9 (4)	C21—O6—C22	115.6 (4)
C7—N1—C1	123.9 (3)	C18—N2—C12	125.1 (2)
C7—N1—H1N	118 (2)	C18—N2—H2N	118 (2)
C1—N1—H1N	118 (2)	C12—N2—H2N	117 (2)
C2—C1—C6	119.1 (3)	C17—C12—C13	119.0 (3)
C2—C1—N1	120.8 (3)	C17—C12—N2	121.7 (3)
C6—C1—N1	120.1 (3)	C13—C12—N2	119.3 (3)
C1—C2—C3	119.8 (3)	C14—C13—C12	119.9 (3)
C1—C2—Cl1	119.6 (2)	C14—C13—Cl3	120.7 (2)
C3—C2—Cl1	120.6 (2)	C12—C13—Cl3	119.5 (2)
C4—C3—C2	120.6 (3)	C15—C14—C13	120.7 (3)
C4—C3—Cl2	118.8 (3)	C15—C14—Cl4	118.9 (3)
C2—C3—Cl2	120.6 (3)	C13—C14—Cl4	120.4 (3)
C5—C4—C3	119.4 (3)	C16—C15—C14	119.3 (3)
C5—C4—H4	120.3	C16—C15—H15	120.4
C3—C4—H4	120.3	C14—C15—H15	120.4
C4—C5—C6	121.1 (3)	C15—C16—C17	120.8 (3)
C4—C5—H5	119.4	C15—C16—H16	119.6
C6—C5—H5	119.4	C17—C16—H16	119.6
C5—C6—C1	119.8 (3)	C16—C17—C12	120.3 (3)
C5—C6—H6	120.1	C16—C17—H17	119.8
C1—C6—H6	120.1	C12—C17—H17	119.8
O1—C7—N1	122.4 (3)	O4—C18—N2	122.9 (3)
O1—C7—C8	122.1 (3)	O4—C18—C19	122.2 (3)
N1—C7—C8	115.5 (3)	N2—C18—C19	114.8 (2)
C7—C8—C9	111.8 (3)	C18—C19—C20	112.9 (3)
C7—C8—H8A	109.3	C18—C19—H19A	109.0
C9—C8—H8A	109.3	C20—C19—H19A	109.0
C7—C8—H8B	109.3	C18—C19—H19B	109.0
C9—C8—H8B	109.3	C20—C19—H19B	109.0
H8A—C8—H8B	107.9	H19A—C19—H19B	107.8
C10—C9—C8	111.9 (3)	C21—C20—C19	113.2 (3)
C10—C9—H9A	109.2	C21—C20—H20A	108.9
C8—C9—H9A	109.2	C19—C20—H20A	108.9
C10—C9—H9B	109.2	C21—C20—H20B	108.9
C8—C9—H9B	109.2	C19—C20—H20B	108.9
H9A—C9—H9B	107.9	H20A—C20—H20B	107.8
O2—C10—O3	124.7 (4)	O5—C21—O6	123.7 (4)
O2—C10—C9	124.3 (4)	O5—C21—C20	125.7 (4)
O3—C10—C9	111.0 (3)	O6—C21—C20	110.6 (3)
O3—C11—H11A	109.5	O6—C22—H22A	109.5
O3—C11—H11B	109.5	O6—C22—H22B	109.5
H11A—C11—H11B	109.5	H22A—C22—H22B	109.5
O3—C11—H11C	109.5	O6—C22—H22C	109.5
H11A—C11—H11C	109.5	H22A—C22—H22C	109.5
H11B—C11—H11C	109.5	H22B—C22—H22C	109.5
			
C7—N1—C1—C2	−133.2 (3)	C18—N2—C12—C17	−45.6 (4)
C7—N1—C1—C6	47.9 (5)	C18—N2—C12—C13	134.9 (3)
C6—C1—C2—C3	1.5 (4)	C17—C12—C13—C14	−0.4 (4)
N1—C1—C2—C3	−177.4 (3)	N2—C12—C13—C14	179.1 (3)
C6—C1—C2—Cl1	−178.1 (2)	C17—C12—C13—Cl3	178.7 (2)
N1—C1—C2—Cl1	3.1 (4)	N2—C12—C13—Cl3	−1.8 (4)
C1—C2—C3—C4	−2.5 (5)	C12—C13—C14—C15	1.0 (5)
Cl1—C2—C3—C4	177.1 (2)	Cl3—C13—C14—C15	−178.1 (3)
C1—C2—C3—Cl2	177.3 (2)	C12—C13—C14—Cl4	−179.9 (2)
Cl1—C2—C3—Cl2	−3.2 (4)	Cl3—C13—C14—Cl4	1.0 (4)
C2—C3—C4—C5	1.4 (5)	C13—C14—C15—C16	−0.7 (5)
Cl2—C3—C4—C5	−178.4 (3)	Cl4—C14—C15—C16	−179.7 (3)
C3—C4—C5—C6	0.7 (5)	C14—C15—C16—C17	−0.2 (6)
C4—C5—C6—C1	−1.7 (5)	C15—C16—C17—C12	0.8 (5)
C2—C1—C6—C5	0.6 (5)	C13—C12—C17—C16	−0.4 (5)
N1—C1—C6—C5	179.4 (3)	N2—C12—C17—C16	−179.9 (3)
C1—N1—C7—O1	6.6 (6)	C12—N2—C18—O4	2.8 (5)
C1—N1—C7—C8	−174.9 (3)	C12—N2—C18—C19	−175.4 (3)
O1—C7—C8—C9	−25.0 (5)	O4—C18—C19—C20	16.9 (5)
N1—C7—C8—C9	156.5 (3)	N2—C18—C19—C20	−164.9 (3)
C7—C8—C9—C10	−60.6 (4)	C18—C19—C20—C21	70.7 (4)
C11—O3—C10—O2	−2.4 (6)	C22—O6—C21—O5	2.2 (7)
C11—O3—C10—C9	178.4 (3)	C22—O6—C21—C20	−178.4 (4)
C8—C9—C10—O2	−6.7 (5)	C19—C20—C21—O5	4.6 (6)
C8—C9—C10—O3	172.5 (3)	C19—C20—C21—O6	−174.7 (3)

### Hydrogen-bond geometry (Å, °)

**Table:**

*D*—H···*A*	*D*—H	H···*A*	*D*···*A*	*D*—H···*A*
N1—H1N···O1^i^	0.85 (1)	2.08 (1)	2.899 (3)	162 (3)
N2—H2N···O4^ii^	0.86 (1)	2.06 (2)	2.880 (3)	159 (3)

Symmetry codes: (i) *x*−1, *y*, *z*; (ii) *x*+1, *y*, *z*.
